# Factors associated with the early termination of exclusive breastfeeding among mother-infant dyads in Samara-Logia, Northeastern Ethiopia

**DOI:** 10.1186/s12887-019-1803-1

**Published:** 2019-11-11

**Authors:** Amanuel Molla Beyene, Misgan Legesse Liben, Amit Arora

**Affiliations:** 1 Forecasting and Capacity Building Officer, Pharmaceuticals Fund and Supply Agency, Dessie Branch, Amhara, Ethiopia; 2 Department of Public Health, College of Health Sciences, Wodia University, Amhara, Ethiopia; 3 School of Science and Health, Western Sydney University, Campbelltown Campus, Locked Bag 1797, Penrith, NSW 2751 Australia; 4 Translational Health Research Institute, Western Sydney University, Locked Bag 1797, Penrith, NSW 2751 Australia; 5 Discipline of Child and Adolescent Health, Sydney Medical School, Faculty of Medicine and Health, The University of Sydney, Westmead, NSW 2145 Australia; 6 Oral Health Services, Sydney Local Health District and Sydney Dental Hospital, NSW Health, Surry Hills, NSW 2010 Australia

**Keywords:** Exclusive breastfeeding, Pastoral, Survival, Afar, Ethiopia

## Abstract

**Background:**

The World Health Organization recommends that mothers should exclusively breastfeed their infants until 6 months of age due to the benefits for the mother and the child. There is documented evidence on exclusive breastfeeding from Ethiopia, but not specifically from Samara-Logia city administration. This study aimed to assess the factors associated with early termination of exclusive breastfeeding among mother-infant dyads in Samara-Logia, Ethiopia.

**Methods:**

A cross-sectional study was conducted in March 2018. Data were collected on 484 randomly selected mother-infant dyads. The Kaplan Meier curve with the log-rank test was used to compare the survival difference. Cox regression models were used to identify the predictors of early termination of exclusive breastfeeding.

**Results:**

The cumulative proportion of survival probability of exclusive breastfeeding to 6 months was 64.5%, with the median duration of 6 months. Mothers having partners with formal education [Adjusted hazard ratio (AHR): 0.58; 95% confidence interval (CI): 0.39, 0.85], receiving counseling on exclusive breastfeeding at antenatal check-up [AHR: 0.62; 95% CI: 0.43, 0.91], giving birth in a health institution [AHR: 0.50; 95% CI: 0.28, 0.88], initiating breastfeeding within the first hour [AHR: 0.41; 95% CI: 0.24, 0.68], and perceiving breast milk adequate for the first 6 months [AHR: 0.17; 95% CI: 0.12, 0.25] were associated with lower hazard of discontinuing exclusive breastfeeding before 6 months.

**Conclusion:**

This study showed that the cumulative proportion of survival probability on exclusive breastfeeding was low in Samara-Logia city administration. Educating husbands to support their partners, strengthening infant feeding counseling, promoting institutional delivery, educating women about the benefit of early initiation of breastfeeding, and expanding urban health extension program are important to improve the duration of exclusive breastfeeding in Ethiopia.

## Background

Breastfeeding provides young infants with the nutrients for growth, development, and health [1]. Human milk is uniquely suited to the infant, both in its nutritional composition and in the non-nutritive bioactive factors, which include cells, anti-infectious, and anti-inflammatory agents and growth factors that promote child survival and healthy child development [2].

International organizations such as the United Nations International Children’s Emergency Fund (UNICEF) and the World Health Organization (WHO) recommend infants should be exclusively breastfed in the first 6 months, and thereafter be given nutritious complementary foods coupled with continued breastfeeding up to the age of 2 years or beyond [3]. Exclusive breastfeeding (EBF) is an infant’s breast milk consumption without supplementation of any type of foods and/or drinks (including water), except for vitamins, minerals and necessary medications up to the age of 6 months [1].

EBF is adequate in quality as well as quantity in terms of the nutrients required by the baby [4]. It eliminates contamination which makes it especially important in resource-poor setting communities [5]. Lack of EBF is associated with infant and childhood morbidity and mortality including lifelong impact on school performance, productivity, and intellectual development [6], and overall health during adolescence and adulthood [7]. Hence, breastfeeding in general, EBF in particular, is among the best interventions in the first 1000 days [8, 9].

Globally, about 40% of infants received exclusively breastfeeding in the first 6 months [10]. In Ethiopia, breastfeeding is nearly universal; about 97% of children are ever breastfeed. However, 58% of infants receive breast milk exclusively with a median duration of 3.1 months. This is lower than the health sector transformation plan of Ethiopia [11], and international recommendations [12].

The government of Ethiopia emphasizes on EBF and has declared ‘the annual exclusive breastfeeding day’ [13]. The health extension program aims at improving proper infant and young child nutrition, for instance, the promotion of EBF in Ethiopia [14]. Besides, nongovernmental organizations are working towards the improvement of optimal breastfeeding in the country [15].

In Ethiopia, particularly in Samara-Logia, there is no adequate evidence on the factors associated with early termination of EBF. Furthermore, health information on child feeding practices is limited among pastoral communities. Therefore, this study aimed to investigate the predictors of EBF duration in Samara-Logia city administration, Afar National Regional State, Ethiopia.

## Methods

### Study setting

A cross-sectional study was employed, in March 2018, on mothers of infants aged 6 up to 12 months in Samara-Logia city administration. The city administration is located at 574 km from Addis Ababa (the capital of Ethiopia). Evidence from Afar National Regional State Health Bureau showed that 57, 285 total population of Samara-Logia. Of which about 13,079 are women in reproductive age group, with 6530 are children aged less than 5 years, and 797 are infants aged 6 to 12 months. There are 13 ketenas (the smallest administrative units next to kebele) in the city administration. There are also two health centers and 13 private clinics.

### Sample size determination

A total of 484 study participants were determined using Open Epi Version 2.3, having the following assumptions: 77.1 and 65.2% magnitude of EBF among women who initiated breastfeeding within the first hour and after the first hour of birth, respectively [14]. Two-sided significance level was set to 95%, power as 80%, and the ratio of sample size (Unexposed/Exposed) =1.

### Sampling procedure and study participants

First, Samara-Logia was purposively selected since there is no evidence on the duration of EBF. Second, all 13 ketenas were included in the study. Thirdly, the total number of study participants was proportionally allocated to all ketenas. Then, simple random sampling was used to select the study participants using a health extension logbook as a sampling frame. However, infants whose mothers were unable to speak, and infants living with non-biological mothers were excluded from the study.

### Data collection process and instrument

Data were collected using an interviewer-administered questionnaire. First, it was developed in English from the Ethiopia demographic and health survey (EDHS) [16] and other literatures [17–20]. Then, the questionnaire was pretested in Dubti town, and the findings were incorporated into the final questionnaire. The final English version of the questionnaire is provided as “Additional file 1” with this article. Finally, the Amharic version was used to collect the data. Six diploma holders in nursing and two public health professionals were recruited as data collectors and supervisors, respectively. Two days training on the data collection procedures was given to data collectors and supervisors.

### Study variables

The outcome variable was the duration of EBF in a month. It was assessed using a ‘since birth’ recall approach. Study participants were asked “What was the age (in months) of this baby (“Name”) when you first tried semi-solids or solids or liquids (including water) other than your breast milk*?*” Then, mothers who exclusively breastfeed their infants to less than 6 months were considered as “events” and those who feed infants to 6 months and beyond were “censored”.

The independent variables were: socio-demographic variables (maternal age, educational status, occupational status, religion, ethnicity, marital status, birth order, number of children, family size, infant’s gender, infant’s age, head of the household, family monthly income), maternal and infant health service-related variables (antenatal check-up (ANC), place and mode of delivery, postnatal check-up (PNC), infant feeding advice at ANC and PNC check-ups, source of information on breastfeeding); and infant feeding (early initiation of breastfeeding, prelacteal feeding, colostrum discarding, bottle feeding, and perceived adequacy of breast milk).

### Data management and analysis

Data were entered using Epi data version 3.02 and exported to SPSS version 20 for statistical analysis. Model fitness was checked by the proportionality hazard assumption test using log (−log) versus log (time) graph and time-dependent Cox model. In both tests, the model was fulfilled.

Descriptive statistics were used to describe the study variables. The Kaplan-Meier survival curve with a log-rank test was used to compare the survival of infants on EBF. The univariable Cox regression model was used to assess the effect of each independent variable on the duration of EBF. Then, variables with *p*-value < 0.25 in the univariable model were included in the final model. In both models, *p*-value < 0.05 was used to declare statistical significance.

## Results

### Characteristics of the study participants

A total of 465 mother-infant dyads participated in the study (the response rate was 96.07%). The mean (+Standard deviation (SD)) of maternal age was 27.72 (+ 4.46) years. Three hundred sixty-two (77.8%) of the respondents were aged less than 30 years, and 411(88.4%) were Muslims by religion. Three hundred seven (66%) were Afar by ethnicity (Table 1).

**Table 1:** Socio-demographic characteristics of mother-infant dyads in Samara-Logia city administration, Afar Regional State, Ethiopia, 2018 (*n* = 465)

Variables	Frequency (n)	Percentage (%)
Maternal age (years)		
< 25	96	20.6
25–34	318	68.4
> 34	51	11.0
Maternal religion		
Christian	54	11.6
Muslim	411	88.4
Ethnicity		
Afar	307	66.0
Amhara	138	29.7
Tigray	17	3.7
Oromo	3	0.6
Maternal educational status		
No formal education	350	75.3
Formal education	115	24.7
Maternal occupation		
Housewife	387	83.2
Other	78	16.8
Maternal marital status		
Living together	447	96.1
Not living together	18	3.9
partner educational status		
No formal education	188	42.1
Formal education	259	57.9
Household head		
Respondent	36	7.7
Husband	429	92.3
Gender of infant		
Male	215	46.2
Female	250	53.8
Age of infant (in months)		
6–8	289	62.2
9–12	176	37.8
Family size		
2	6	1.3
3–4	140	30.1
> 5	319	68.6
Average monthly income (ETB)		
< 1000	51	11.0
> 1000	414	89.0
Birth order		
1	69	14.8
2–4	259	55.7
> 4	137	29.5

*ETB* Ethiopian Birr

### Maternal and infant health service utilization

Four hundred sixty (98.9%) of the study mothers had attended at least one antenatal (ANC) check-up. Of mothers who had received ANC check-up, about 29% had received counseling on infant feeding (Table 2).

**Table 2:** Maternal and infant health service utilization in Samara-Logia city administration, Afar Regional State, Ethiopia, 2018 (*n* = 465)

Variables	Frequency (n)	Percentage (%)
ANC checkup^a^		
Yes	460	98.9
No	5	1.1
Frequency of ANC visits		
1	26	5.7
2–3	269	58.5
> 4	165	35.9
Infant feeding counseling during ANC		
Yes	329	71.5
No	131	28.5
Place of birth		
Home	34	7.3
Health institution	431	92.7
Mode of delivery		
Cesarean section	29	6.2
Vaginal	436	93.8
PNC checkup^a^		
Yes	268	57.6
No	197	42.4
Infant feeding counseling during PNC		
Yes	242	90.3
No	26	9.7
Source of infant feeding Information		
HEWs	309	66.5
Others	156	33.5

*ANC* Antenatal care, *PNC* postnatal care, *HEWs* health extension workers

^a^at least one checkup

### Infant feeding practices

Four hundred sixty-four (99.8%) of the respondents had breastfed their infants at any point in time, with about 87.7% (*n* = 407) initiated breastfeeding within the first hour of birth. Three hundred ninety-one mothers (84.1%) believed that breastfeeding is enough for the first 6 months (Table 3). The cumulative survival probability of EBF to 6 months was 64.5%, with a median duration of 6 months. In addition, the range of EBF duration was from 0 to 6 months, respectively (Table 4).

**Table 3:** Infant feeding practices in Samara-Logia city administration, Afar Regional State, Ethiopia, 2018 (*n* = 465)

Variables	Frequency (n)	Percentage (%)
Ever breastfeeding		
Yes	464	99.8
No	1	0.2
Early initiation of breastfeeding		
Yes	407	87.7
No	57	12.3
Prelacteal feeding		
Yes	70	15.1
No	394	84.9
Colostrums discarding		
Yes	56	12.0
No	409	88.0
Current breastfeeding		
Yes	456	98.1
No	9	1.9
Perceived adequacy of breast milk		
Yes	391	84.1
No	74	15.9
Bottle feeding		
Yes	192	41.3
No	273	58.7

**Table 4:** Life table for exclusive breastfeeding duration to the first 6 months among mothers of infants aged 6–12 months in Samara-Logia city administration, Afar Regional State, Ethiopia, 2018(*n* = 465)

Interval start time	Number entering interval	Number of terminating censored	Proportion surviving (%)	Cumulative proportion surviving at end of interval (%)	Proportion of censored (%)	Cumulative censored
0	465	71	85	84.7	15.3	71
1	394	1	99.7	84.5	15.5	72
2	393	4	99	83.7	16.3	76
3	389	18	95	79.8	20.2	94
4	371	39	89	71.4	28.6	133
5	332	32	90	64.5	35.5	165
6	300	(event)	100	64.5	35.5	165

### Factors affecting the duration of exclusive breastfeeding

**Fig. 1:**
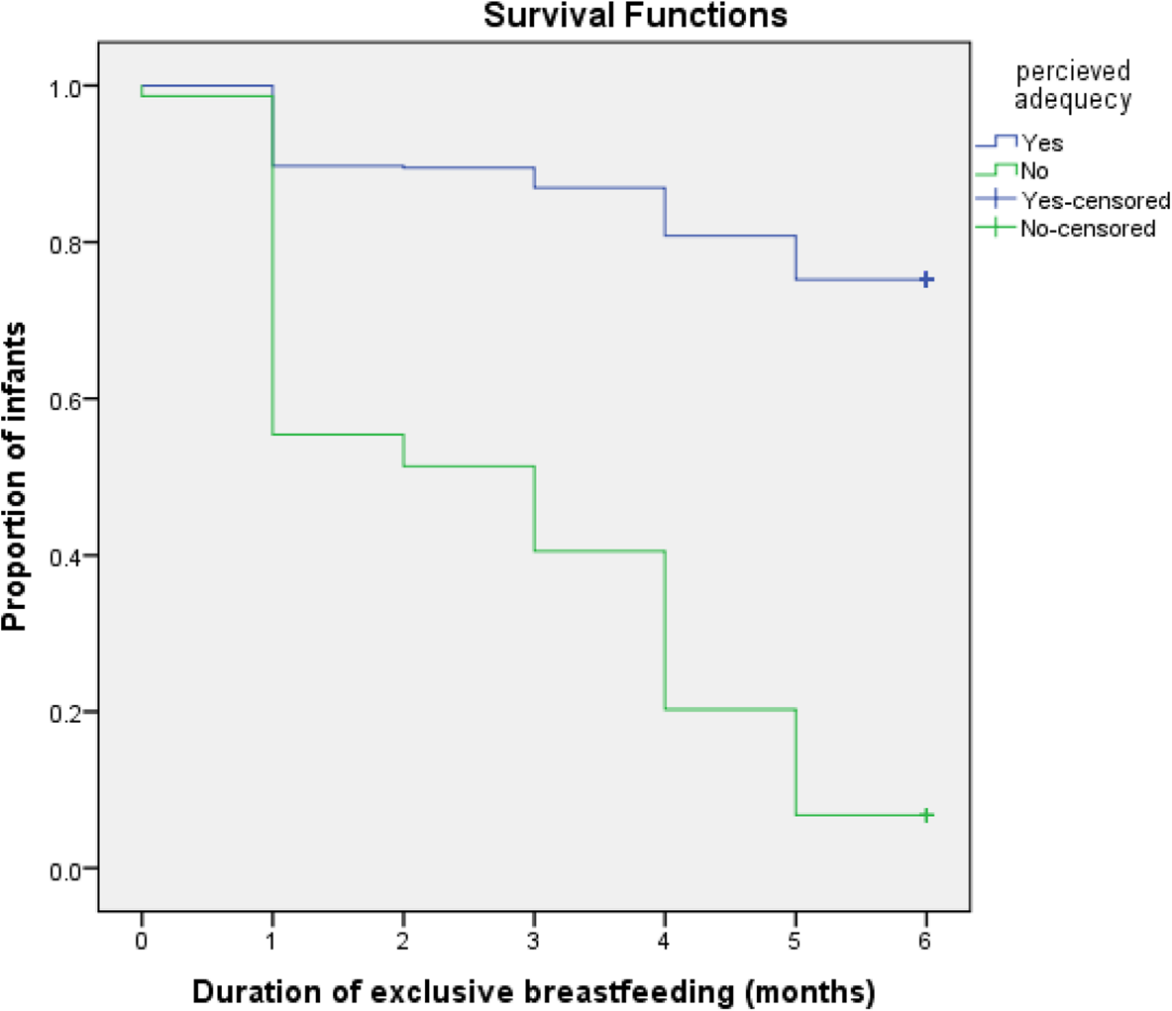
Cumulative Survival probability of exclusive breastfeeding practice in relation to women’s perception on adequacy of their breast milk, Samara-Logia city administration, Afar National Regional State, Ethiopia, 2018 (log rank test < 0.001)

**Fig. 2:**
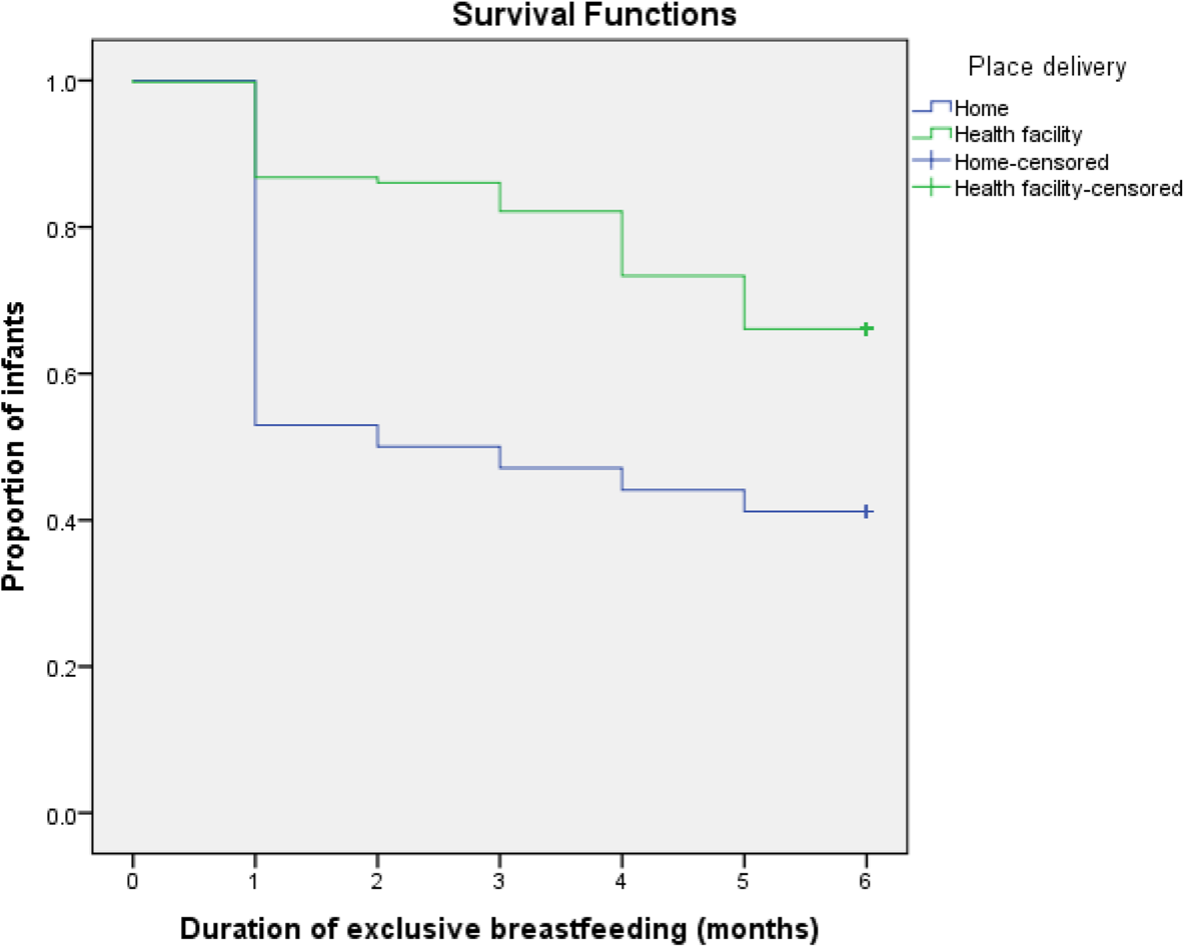
Cumulative Survival probability of exclusive breastfeeding practice in relation to place of delivery, Samara-Logia city administration, Afar National Regional State, Ethiopia, 2018 (log rank test < 0.05)

**Fig. 3:**
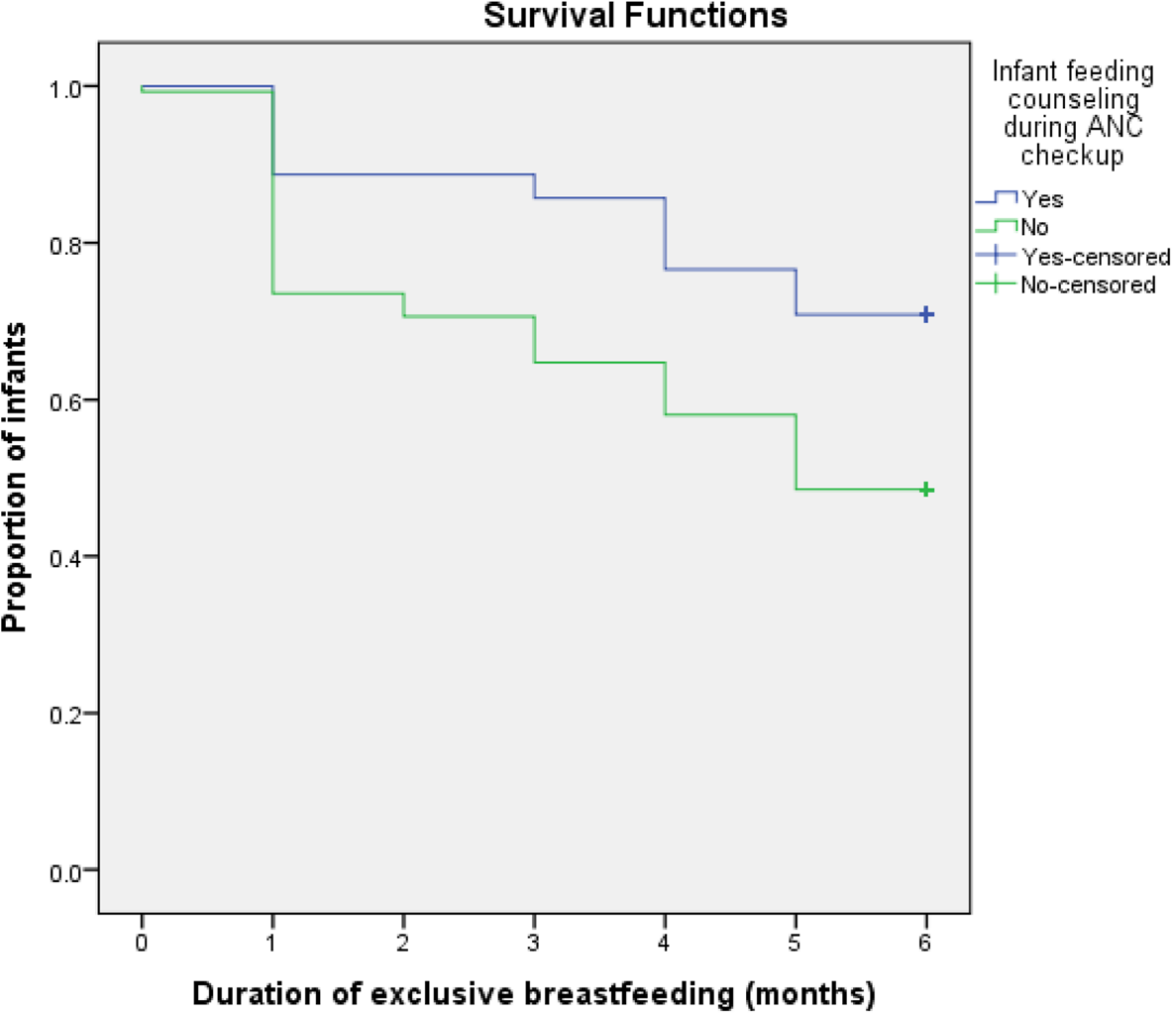
Cumulative Survival probability of exclusive breastfeeding practice in relation to infant breastfeeding counseling at ANC check-up, Samara-Logia city administration, Afar National Regional State, Ethiopia, 2018 (log rank test < 0.05)

Kaplan-Meier curve showed that women’s perceived adequacy of breast milk significantly affected the duration of EBF. The survival curve of women who perceived adequacy of their breast milk was constantly above the survival curve of the other group (log-rank test, *p* < 0.001) (Fig. 1). Women who gave birth in a health institution were more likely to exclusively breastfeed their infants to 6 months as compared to those who gave birth at home (log-rank test, *p* < 0.05) (Fig. 2). The survival probability of EBF was significantly higher among women who had received infant feeding counseling at ANC check-up as compared to those who had deprived of counseling (log-rank test, *p* < 0.05) (Fig. 3**)**.

Univariable Cox regression analysis showed that maternal education, maternal occupation, partner’s education, infant feeding counseling at ANC check-up, attending postnatal check-up (PNC), early initiation of breastfeeding, perceived adequacy of breast milk, mode of delivery, place of delivery, and colostrum avoidance were significant at *p* < 0.25. In the multivariable Cox regression model partner’s education, infant feeding counseling at ANC check-up, place of delivery, early initiation of breastfeeding, and perceived adequacy of breastmilk were statistically significant at *p* < 0.05 (Table 5).

**Table 5:** Univariable and multivariable cox regression model on predictors of early cessation of exclusive breastfeeding among mothers of infants aged 6–12 months in Samara-Logia city administration, Afar Regional State, Ethiopia, 2018

Variable	Early cessation of EBF		CHR (95% Cl)	AHR(95% Cl)
	Yes n(%)	No n(%)		
Maternal educational status				
Non formal	134 (38.3)	216 (61.7)	1.54 (1.04, 2.28)^*^	1.24 (0.74, 2.06)
Formal	31 (27.0)	84 (73.0)	1	1
Maternal occupation				
Housewife	142 (36.7)	245 (63.3)	1.28 (0.82, 1.98)	1.09 (0.60, 1.99)
Other	23 (29.5)	55 (70.5)	1	1
Partner’s educational status				
Non formal	90 (47.9)	98 (52.1)	1	1
Formal	66 (25.5)	193 (74.5)	0.44 (0.32, 0.60)^*^	0.58 (0.39, 0.85)^*^
Infant feeding counseling during ANC checkup				
Yes	96 (29.2)	233 (70.8)	0.49 (0.36, 0.68)^*^	0.62 (0.43, 0.91)^*^
No	67 (51.1)	64 (48.9)	1	1
Place of delivery				
Home	20 (58.8)	14 (41.2)	1	1
Health- institution	145 (33.6)	286 (66.4)	0.43 (0.27, 0.69)^*^	0.50 (0.28, 0.88)^*^
Mode of delivery				
Cesarean section	17 (58.6)	12 (41.4)	1	1
Vaginal	148 (33.9)	288 (66.1)	0.49 (0.30, 0.81)^*^	0.80 (0.39, 1.60)
PNC checkup				
Yes	87 (32.5)	181 (67.5)	0.78 (0.57, 1.05)	1.09 (0.76, 1.56)
No	78 (39.6)	119 (60.4)	1	1
Early initiation of breastfeeding				
Yes	130 (31.9)	277 (59.6)	0.49 (0.34, 0.72)^*^	0.41 (0.24, 0.68)^*^
No	34 (68.1)	23 (40.4)	1	1
Discarding of colostrum				
Yes	27 (48.2)	29 (51.8)	1	1
No	138 (33.7)	271 (66.3)	0.62 (0.41, 0.94)^*^	1.57 (0.92, 2.68)
Perceived adequacy of breast milk				
Yes	96 (24.6)	295 (75.4)	0.16 (0.12, 0.22)^*^	0.17 (0.12, 0.25)^*^
No	69 (93.2)	5 (6.8)	1	1

*ANC* Antenatal care, *PNC* Postnatal Care, *CHR* Crude Hazard Ratio, *AHR* Adjusted Hazard Ratio

^*^Significant at *p* < 0.05

Women whose partners had attended formal education were less likely to discontinue EBF before 6 months as compared to those without formal education [AHR: 0.58; 95% CI: 0.39, 0.85]. Infant feeding counseling at ANC check-up was positively associated with EBF duration. Mothers who received counseling on infant feeding at ANC check-up were less likely to discontinue EBF before 6 months as compared to those who did not receive counseling [AHR: 0.62; 95% CI:0.43, 0.91].

Mothers who gave birth in a health institution were less likely to terminate EBF before 6 months as compared to those who gave birth at home [AHR: 0.50; 95% CI:0.28, 0.88]. Women who initiated breastfeeding within the first hour of birth were less likely to cease EBF as compared to those who had initiated lately [AHR: 0.41; 95% CI: 0.24, 0.68]. Women who reported adequacy of their milk were less likely to terminate EBF early as compared to those who perceived inadequate [AHR: 0.17, 95% CI: 0.12, 0.25] (Table 5).

## Discussion

This study revealed that the median duration of EBF was 6 months which is similar to the international recommendation [3]. The cumulative survival probability of EBF to 4 months and 6 months was declined by 13 and 20%, respectively, from birth. In Ethiopia, traditional postpartum care is given at home by their family members in the first 6 to 12 weeks after delivery [21]. This condition increases the likelihood of mothers and infants to stay together at home which might decrease the hazard of early termination of EBF. In addition, the current study showed that the cumulative survival probability of EBF to 6 months was 64.5%. This finding is lower than the findings from previously conducted studies in Ethiopia [22–24]. This difference may be due to the age of the study participants.

In this study, partner educational status significantly affected the duration of EBF. Women whose partners had attended formal education were at lower hazard of terminating EBF as compared to those who had no formal education. This may be partners without formal education might enforce their wives to give additional foods early as compared to those with formal education. Similar findings were found in Gondar town where women who receive social support were more likely to practice EBF as compared to those without support [13]. This is also consistent with the finding in Australia [25, 26].

This study revealed that counseling on infant feeding at ANC check-up was significantly associated with the duration of EBF. Women who received counseling on infant feeding at ANC check-up were less likely to terminate EBF compared to those who did not receive counseling. This finding is consistent with the previous studies in Ethiopia [18, 19, 27], and Tanzania [28]. This shows that antenatal check-up is an appropriate time to provide essential messages about proper infant feeding practices. Furthermore, the initiation of an urban health extension program in Afar has a great contribution to the access of ANC services including breastfeeding counseling.

Women who delivered a baby in health facilities were less likely to cease EBF as compared to those who delivered a baby at home. Similar findings were reported from different parts of Ethiopia [29–32]. This may be explained in such a way that institutional delivery provides a favorable environment for the early initiation of breastfeeding. This is a key factor that could favor exclusive breastfeeding.

Furthermore, early initiation of breastfeeding was associated minimum hazard of EBF termination as compared to late initiation of breastfeeding. This finding is consistent with the previous Ethiopian studies [17, 20, 23, 27, 33]. This is because initiating breastfeeding within 1 h may lead to increased newborn-mother bonding and sufficient breast milk secretion. This may, in turn, lead to late initiation of additional foods other than breast milk.

Compared to women who perceived inadequacy of breast milk for the first 6 months, those who perceived adequate were less likely to cease EBF. In line with this finding, in the Gurage zone, mothers who perceived adequacy of breast milk were less likely to cease EBF earlier as compared to those who perceived inadequate [24].

The study could be subjected to recall bias. Besides, the study is conducted in the urban area, therefore, might not be a true reflection of the entire Afar community.

## Conclusions

The cumulative survival probability of EBF to 6 months was 64.5%. Women whose partners had formal education, received counseling on infant feeding at ANC check-up, gave birth in a health institution, initiated breastfeeding early, and perceived breast milk adequate for the first 6 months were less likely to terminate EBF before 6 months. Therefore, health promotion interventions should be targeted at educating fathers and involve them in breastfeeding decisions. It is also important to encourage fathers to support their partners and participate in ANC check-ups. Furthermore, strengthening infant feeding counseling both at community and institution level, encouraging institutional delivery, and educating mothers about the importance of early initiation of breastfeeding is important to improve the duration of EBF in pastoralist communities of Ethiopia.

## Supplementary information

**Additional file 1.** Questionnaire to assess factors associated with the early termination of exclusive breastfeeding.

## Data Availability

The findings were declared from the available data sources. All possible required information is included in the manuscript. In addition, the data are available from the corresponding author.

## Abbreviations

AHR: Adjusted hazard ratio
ANC: Antenatal check-up
CI: Confidence interval
EBF: Exclusive breastfeeding
EDHS: Ethiopia demographic and health survey
PNC: Postnatal check-up
RERC: Research Ethics Review Committee
SD: Standard deviation
SPSS: Statistical package for the social sciences
UNICEF: United Nations International Children’s Emergency Fund
WHO: World Health Organization
