# Supplementary material for: Factors associated with the early termination of exclusive breastfeeding among mother-infant dyads in Samara-Logia, Northeastern Ethiopia
Source: BMC Pediatr. 2019 Nov 11;19:428. doi: 10.1186/s12887-019-1803-1 (PMC6844048; doi:10.1186/s12887-019-1803-1)
Supplement: Supplementary file 1 — Additional file 1. Questionnaire to assess factors associated with the early termination of exclusive breastfeeding. [file 12887_2019_1803_MOESM1_ESM.docx]

| **Part 1: Socio-demographic characteristics of study participants** | | |  |
| --- | --- | --- | --- |
| **S.no** | **Questions** | **Categories** | **Skip** |
| **101** | What is your age? | _____________ years |  |
| **102** | Residence? | 1. Rural 2. Urban |  |
| **103** | What is your religion? | 1. Orthodox 2. Muslim 3. Protestant 4. Others (specify)_______ |  |
| **104** | What is your Ethnicity? | 1. Afar 2. Amhara 3. Tigray 4. Other (specify) |  |
| **105** | What is your educational level? | 1. Illiterate 2. Able to read and write 3. Primary (1-8) 4. Secondary(9-12) 5. Higher Education institution (12+) 6. Other (specify) |  |
| **106** | What is your current occupational status? | 1. House wife 2. Farmer 3. Merchant 4. Government employee 5. Daily laborer 6. Other (specify) |  |
| **107** | What is your marital status? | 1. Single 2. Married 3. Divorced 4. Widowed |  |
| **108** | What is your husband’s educational level? | 1. Illiterate 2. Able to read and write 3. Primary (1-8) 4. Secondary(9-12) 5. Higher Education institution (12+) 6. Other (specify) |  |
| **109** | Who is the household head? | 1. Respondent 2. Husband 3. Respondent and husband 4. Other (specify) |  |
| **110** | How many children do you have? | ________ (number) |  |
| **111** | What is the sex of the index child? | 1. Male 2. Female |  |
| **112** | What is the age of the index child? | ___________ months |  |
| **113** | What is the birth order of the index child? | ___________ th |  |
| **114** | What is your family size? | ___________ individuals |  |
| **115** | What is the average monthly income of the house hold? | ___________Ethiopian birr |  |

| **Part 2: Maternal and child health related factors** | | | | |  |
| --- | --- | --- | --- | --- | --- |
| **S.no** | | **Questions** | | **Categories** | **Skip** |
| **201** | | Have you attended Antenatal care while you were pregnant with this child? | | 1. Yes 2. No | If 2 skip to 205 |
| **202** | | Where did you receive antenatal care for this child?  (If unable to determine if public or private sector, write the name of the place?) | | 1. Home 2. Govt. health institution 3. private health institution 4. Other (specify) |  |
| **203** | | How many times did you receive antenatal care for this child during? (*If the respondent do not know, please select the option “do not know*” ) | | Number of visits ____________  Do not know |  |
| **204** | | During (any of) your antenatal care visit(s), were you advised about exclusive breastfeeding? | | 1. Yes 2. No 3. Do not know |  |
| **205** | | Where did you give birth to the child? | | 1. Home 2. Govt. health institution 3. private health institution 4. Other (specify) |  |
| **206** | | What was the mode of delivery? | | 1. Caesarean section 2. Vaginal delivery |  |
| **207** | | After you gave birth to the child, have you attend any post natal care visit(s)? | | 1. Yes 2. No | If 2 skip to 209 |
| **208** | | During your post natal care visit, were you advised about proper exclusive breastfeeding practices? | | 1. Yes 2. No 3. Do not know |  |
| **209** | | What are your information sources for infant feeding practices? | | 1. Radio 2. Television 3. Husband 4. Mother 5. HEW 6. Nurse 7. Doctor 8. Other (specify) |  |
|  | | | | |  |
| **Part 3: Infant feeding practices** | | | | |  |
| **s.no** | **Questions** | | **Categories** | | **Skip** |
| **301** | Did you ever breastfeed this child child? | | 1. Yes 2. No | | If 2 skip to 306 |
| **302** | How long after birth did you put a baby on breast? (If less than 1 hour record ‘Immediately after birth'. If less than 24 record “hours”. Otherwise, record “days”.) | | Immediately after birth  Hours……………..  Days.…………….. | |  |
| **303** | Was the infant given fluid or feeds before the initiation of breastfeeding? | | 1. Yes 2. No | | If 2 skip to 305 |
| **304** | What was given to drink and/or to eat?  (Record all mentioned). | | 1. Fresh butter 2. Honey 3. Milk other than mother’s milk 4. Plain water 5. Other (specify) | |  |
| **305** | Do you know what colostrum is? | | 1. Yes, first yellow milk 2. No 3. Other (specify) | |  |
| **306** | What did you do with the first milk (Colostrum)? | | 1. Throw away 2. Give to child | | If 2 skip to 309 |
| **307** | What was the reason not to give colostrum to the infant? | | 1. Not good for the infant 2. It is the tradition 3. It was yellow / thick 4. It seems useless 5. It is dirty 6. Infant unable to feed 7. Influenced by some one 8. Other (specify) | |  |
| **308** | If you influenced by others, who influenced you to discard colostrum? | | 1. Husband 2. Religious leader 3. Traditional birth attendant 4. Health extension workers 5. My mother 6. Health professional 7. Other (specify) | |  |
| **309** | What is the importance of colostrum for the new born?  (Do not read the choices.) | | 1. First immunization 2. Child growth 3. Do not know 4. Other (specify) | |  |
| **310** | Are you still breastfeeding the child? | | 1. Yes 2. No | |  |
| **311** | What was the age (in months) of this baby (“Name”) when you first tried semi-solids or solids or liquids (including water) other than your breast milk*?* | | _______________ month(s) | |  |
| **312** | If you influenced by others, who influenced you to give other foods or drinks before six months? | | 1. Husband 2. Religious leader 3. Traditional birth attendant 4. Health extension workers 5. My mother 6. Health professional 7. Other (specify) | |  |
| **313** | Do you believe that breast milk is enough for the first six months? | | Yes  No | |  |
| **314** | Did the child drink anything from a bottle with a nipple yesterday or last night? | | 1. Yes 2. No | |  |
| **315** | Did the child eat any solid, semi-solid, or soft foods yesterday during the day or at night? | | 1. Yes 2. No | |  |
